# Effects of Selenium Supplement on B Lymphocyte Activity in Experimental Autoimmune Thyroiditis Rats

**DOI:** 10.1155/2021/9439344

**Published:** 2021-08-10

**Authors:** Yang Li, Xinhe Zuo, Chuan Hua, Yong Zhao, Xun Pei, Man Tian

**Affiliations:** ^1^Thyroid Center of Hubei Provincial Hospital of Traditional Chinese Medicine, Wuhan, 430070, China; ^2^Hubei Province Academy of Traditional Chinese Medicine, Wuhan 430074, China; ^3^Hubei University of Traditional Chinese Medicine, Wuhan 430061, China

## Abstract

**Methods:**

45 healthy and adult female SD rats were randomly divided into three groups: normal control group, EAT model group, and selenium yeast supplement EAT group. The EAT model rats were induced by subcutaneous injection of porcine thyroglobulin and fed with high iodine water. The concentrations of serum thyroid-stimulating hormone (TSH), TGAb, TPOAb, and B cell activating factor (BAFF) were detected in each group by enzyme-linked immunosorbent assay (ELISA), and the expression of interleukin-10 (IL-10) in thyroid tissue was detected by immunohistochemistry. B cells and regulatory B cells (Bregs) ratios in the spleen of rats were analyzed by flow cytometry.

**Results:**

In contrast with the EAT model group, the levels of serum TSH, TGAB, TPOAb, and BAFF were decreased, while IL-10 expression was increased in thyroid tissue, and Bregs ratio was upregulated in the spleen (all *p* < 0.05) in the selenium yeast supplement EAT group.

**Conclusion:**

Selenium yeast supplement could partially attenuate immune imbalance in EAT rats, which may be related to the mechanism of modulating B lymphocyte activity.

## 1. Introduction

Autoimmune thyroid diseases (AITD) are the most common autoimmune disorders, and autoimmune thyroiditis (AIT) is the most frequent AITD, which may lead to pathological changes in the thyroid, such as lymphocytes infiltration, fibrosis, and swelling [1, 2]. AIT is related to hypothyroidism, thyroid nodules, and even thyroid malignancies, such as papillary thyroid carcinoma [3, 4]. Additionally, it may have adverse effects on pregnancy outcome [5, 6], which raises more and more attention around the world. Therefore, it is of importance to explore the prevention and treatment of AIT. Currently, to the best of our knowledge, the efficacy of the existing therapies for AIT is uncertain.

It is considered that autoantibodies to thyroid peroxidase (TPO) and thyroglobulin (TG) are crucial components in AIT pathogenesis. TPOAb and TGAb are produced by B lymphocytes. Regulatory B cells (Bregs) have been identified related to decrease the production of proinflammatory cytokines [7, 8], and the defective expression of Bregs might play an important role in the development of AIT [9]. Regulation of B cell activity is a potential target to treat AIT.

Selenium is a micronutrient of diet with many pleiotropic effects ranging from antioxidant to anti-inflammatory [10] and is also an essential micronutrient that plays a crucial role in the immune system [11]. Selenium is often prescribed in the treatment of AIT, while the effects of selenium supplementation on AIT were controversial. Some reports have confirmed a suppressive effect of selenium supplementation on serum TPOAb and TGAb levels in AIT patients [12, 13]. However, an RCT study concluded that selenite supplementation had no effect on serum TPOAb and TSH levels in euthyroid TPOAb-positive women [14]. Other studies showed selenium could prevent apoptosis of thyroid follicular cells [15, 16]. Moreover, the mechanism of how selenium regulates B cell activity in AIT also remains uncertain. Therefore, the present study aimed to investigate the action of organic selenium (selenium yeast) supplementation on thyroid autoimmunity and B and Breg lymphocyte activity in iodine-induced experimental autoimmune thyroiditis (EAT) rats.

## 2. Materials and Methods

### 2.1. Main Reagents and Instruments

Porcine thyroglobulin (PTG), sodium iodide (NAI), complete Freund's adjuvant (CFA), and incomplete Freund's adjuvant (IFA) were purchased from Sigma-Aldrich Company (t1126, 746371, f5881, and f5506, respectively). B cell activating factor (BAFF) detection kit was from USCN KIT INC (seb686ra), while the Dako real envision detection system was from Agilent Dako (k5007). Enzyme-linked immunosorbent assay (ELISA) kit for thyroid stimulating hormone (TSH), thyroglobulin antibody (TGAb), and thyroid peroxidase antibody (TPOAb) was obtained from IRET company (e-el-r0976, sbj-r0551, and csb-e11199r, respectively). Main antibodies were as follows: IL-10 rabbit anti-mouse (ABclonal company, a2171), CD45RA FITC mouse anti-rat (BD Bioscience, 561886), and IL-10 PE mouse anti-rat (BD Bioscience, 555088).

Main instruments are BIO-DAD 680 enzyme mark instrument, flow cytometry (Beckman, CytoFLEX), microscope (Olympus, Japan), and HMIAS-2000 image analysis system (HMIAS, China).

### 2.2. Animals and Diet

45 healthy and adult female SD rats, weighing about 100–120 g, were obtained from Hubei Experimental Animal Center, China (animal license SCXK 2017–0012, certificate 42010200002809). All experimental procedures were carried out in accordance with standard guidelines for the care of laboratory animals of Hubei University of Traditional Chinese Medicine and were approved by the ethics committee for research on laboratory animal use of the institution. The rats were reared in a specific pathogen-free condition, room temperature 16–24°C, humidity 40–60%, and adaptive ordinary feeding for one week.

### 2.3. Preparation of the EAT Model and Treatments

EAT model rats were induced according to previous studies by immunization with thyroglobulin and fed with high iodine water [17–20]. The rats were randomly divided into three groups (normal control, N; EAT model group, M; selenium yeast supplement EAT group, Se), and each group consisted of 15 rats. The N group was fed with a normal diet throughout the entire experiment. The rats of M group and Se group were immunized with PTG thrice and given high iodine water (preparation of high iodine water: 0.64 g sodium iodide into 1 L water) until the end of the experiment. The EAT model inducing procedure was as follows: PTG (2 mg/ml in PBS) was emulsified (1 : 1) with complete Freund's adjuvant (CFA, Sigma), and 100 ug PTG emulsion (1 mg/ml in CFA) was injected subcutaneously at multiple sites of the hind legs of each rat on 1st day and 7th day. After first immunized, 100 ug PTG emulsion in incomplete Freund's adjuvant (IFA, Sigma) (1 mg/ml in IFA) was given to rats, respectively, as a booster immunization, on 14th day, 21st day, 28th day, and 35th day. After last immunization, rats in the Se group were given selenium yeast saline solution (containing Se 18 *μ*g•kg^−1^) by gavage, twice per day. The Se dosage was determined by the rational comparison of drug doses in experimental animals and humans [21] (3 *μ*g•kg^−1^ selenium yeast was prescribed to AIT patients in clinics, orally taken, twice per day). The rats in *N* and M groups were treated with normal saline. All gavage treatments lasted 8 weeks.

### 2.4. Serological Assays

After 8 weeks of the above treatments, diets were removed from the cages 12 h before the rats were sacrificed. Serum samples were collected from each group of rats, and the levels of serum TSH, TGAb, TPOAb, and BAFF were assayed by ELISA (as previous studies reported [22, 23]), according to the manufacturers' instructions.

### 2.5. Thyroid Histopathology

After all animals were sacrificed, thyroids were surgically removed, put in a buffer solution of 10% formalin, and embedded in paraffin. 5 *μ*m thick sections were prepared and stained with hematoxylin and eosin (HE). Histological changes in the thyroid tissue were observed. Thyroid histopathology scores were based on the percentage of thyroid follicles infiltrated with lymphocytes as previous study described [9, 18], and the severity of thyroiditis was graded on a scale of 0–4: grade 0, normal; grade 1, 1–10%; grade 2, 10–30%; grade 3, 30–50%; and grade 4, more than 50%.

IL-10 expression in the thyroid was examined by immunohistochemistry using a primary IL-10 antibody (1 : 200 dilution) incubated overnight at 4 C, followed by incubation with a species-specific secondary antibody for 30 min at 37°C and staining with diaminobenzidine. Five discontinuous sections from each rat were stained. The images (at 400× magnification) were captured, and the absorbance was analyzed using the HMIAS-2000 high-definition color medical analysis system [24].

### 2.6. Flow Cytometry

Spleens were surgically removed from each group of rats. Single cell suspensions of splenocytes were prepared, and immunophenotyping analysis of B cell and Breg cell was conducted according to previous study [25, 26]. CD45RA positive cells were B cells, while Breg cells were positive on both CD45RA and IL-10. For B cell staining, splenocytes from single cell suspensions (1 × 10^6^ cells/ml) were added with FITC-labeled anti-CD45RA antibody and incubated at 4°C for 30 min in dark; after centrifugation at 2000 rpm for 3 min, the cells were washed once with phosphate buffer saline (PBS) containing 0.5% bovine serum albumin (BSA) and then resuspended with 0.2 ml PBS containing 0.5% BSA and analyzed by flow cytometry. For analysis of Breg cell, isolated spleen cells of each group were inoculated with 1 × 10^6^ cells in a 24-well plate, stimulated with phorbol ester (50 ng/ml), ionomycin (1 *μ*g/ml), and monensin (2 *μ*M), and incubated for 6 h in 5% CO_2_ at 37°C; then, the cells were stained with FITC-labeled anti-CD45RA antibody and PE-labeled anti-IL-10 antibody successively and finally analyzed by flow cytometry.

### 2.7. Statistical Analysis

The statistical analyses were carried out with SPSS version 24.0. All results were shown as the mean ± SEM. Data were analyzed by analysis of variance with Bonferroni's post hoc test. Statistical significance was defined as *p* < 0.05.

## 3. Results

### 3.1. Selenium Yeast Supplement Decreased the Levels of Serum TSH, TGAb, and TPOAb in EAT Rats

**Figure 1:**
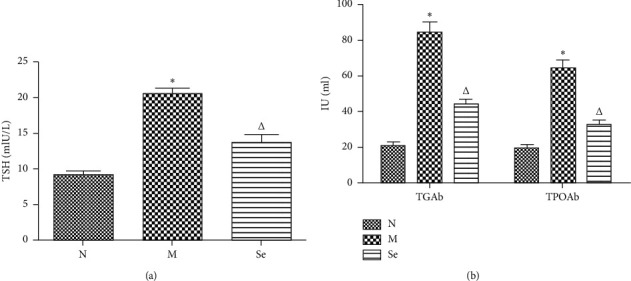
(a) Serum TSH titers in each group rats. (b) Serum TGAb and TPOAb titers in each group rats. N, normal control; M, EAT model group; Se, selenium yeast supplement EAT group. ^*∗*^*P* < 0.05 compared to the N group. ^△^*P* < 0.05 compared to the M group.

As Figures 1(a) and 1(b) show, the serum TSH as well as TGAb and TPOAb titers were significantly elevated in the M group rats compared to the N group rats. In the selenium yeast supplement EAT group (Se), we observed that serum TSH, TGAb, and TPOAb titers were obviously decreased, which was in contrast to the EAT model group (M).

### 3.2. Selenium Yeast Supplement Decreased the Infiltration of Lymphocytes in the Thyroid

**Figure 2:**
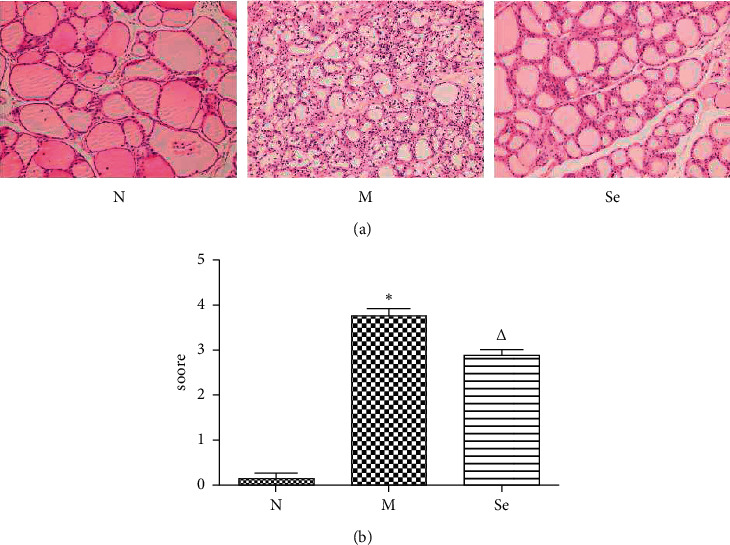
(a) Representative histographs of H&E-stained thyroid sections. (b) Thyroid histopathology scores. ^*∗*^*P* < 0.05 compared to the N group. ^△^*P* < 0.05 compared to the M group (at 200× magnification).

As Figure 2 shows, a number of lymphocytes infiltrated in the thyroid of the M group rats, and histopathology scores increased dramatically compared with the N group rats. In addition, a partial of thyroid follicular structure was abnormal, indicating the incidence of thyroiditis, which was in accordance with previous studies [9, 20]. Selenium yeast supplement significantly relieved lymphocytes infiltration in thyroid as we observed in the Se group rats in contrast with the M group rats.

### 3.3. Selenium Yeast Supplement Lowered the Serum BAFF Level in EAT Rats

**Figure 3:**
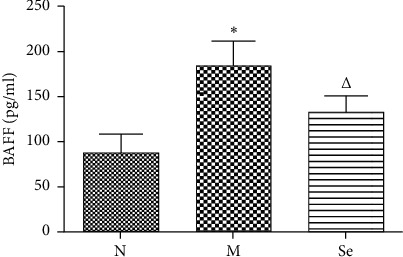
Serum BAFF level detected by ELISA. ^*∗*^*P* < 0.05 compared to the N group. ^△^*P* < 0.05 compared to the M group.

As Figure 3 shows, the serum BAFF level increased in the M group rats in contrast with the N group, while selenium yeast supplement lowered the serum BAFF level in the Se group rats compared with the M group rats.

### 3.4. Selenium Yeast Supplement Increased IL-10 Expression in the Thyroid of EAT Rats

**Figure 4:**
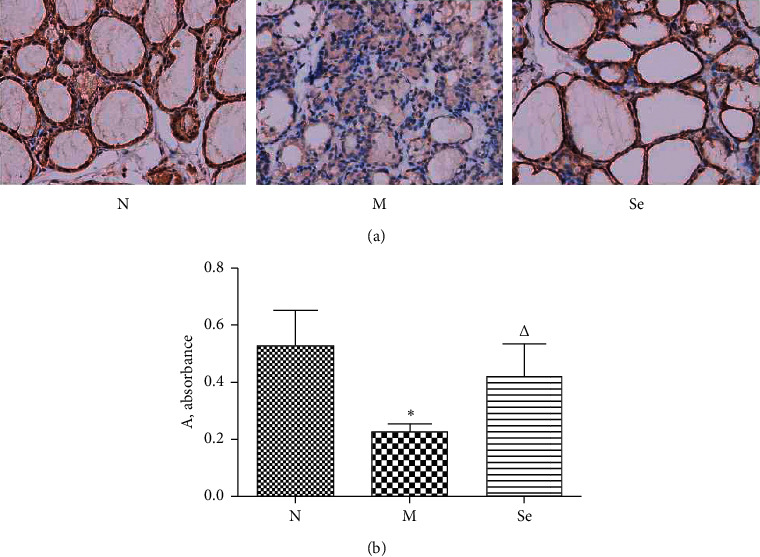
(a) Representative histographs of IL-10-stained thyroid sections. (b) Semiquantitative analysis of IL-10 expression in the thyroid, by absorbance evaluation. ^*∗*^*P* < 0.05 compared to the N group. ^△^*P* < 0.05 compared to the M group (at 400× magnification).

As shown in Figure 4, IL-10 expression in the thyroid of EAT rats in the M group was suppressed in contrast with the N group. After selenium administration, IL-10 expression significantly elevated in the thyroid of the Se group compared with the M group.

### 3.5. Selenium Yeast Supplement Enhanced B Cell and Breg Cell Ratios in EAT Rats

**Figure 5:**
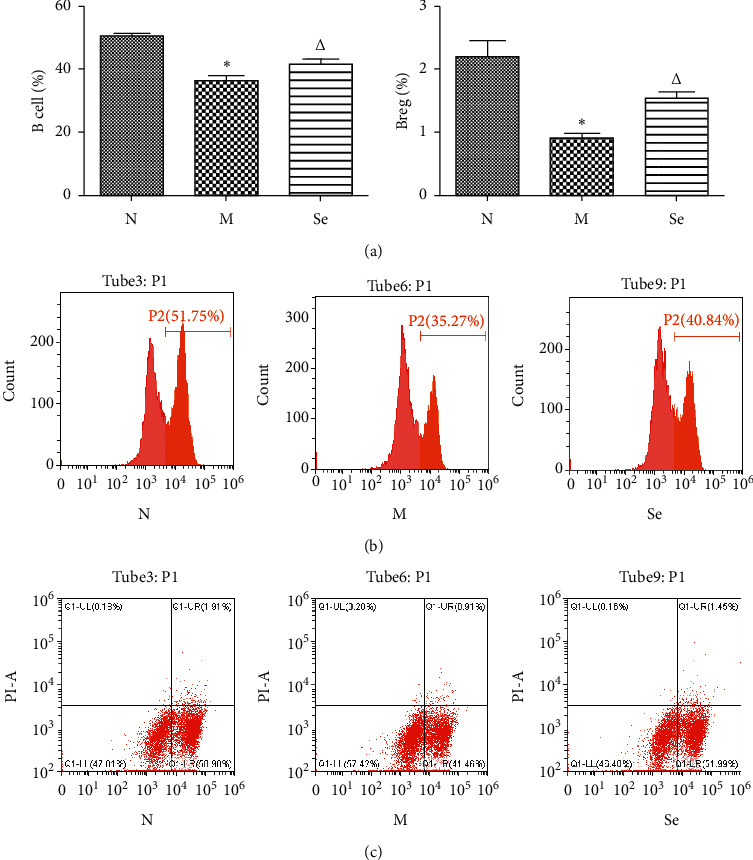
(a) B cell and Breg cell ratios in the spleen tested by flow cytometry. ^*∗*^*P* < 0.05 compared to the N group. ^△^*P* < 0.05 compared to the M group. (b) Representative images of flow cytometry analysis of B cell. (c) Representative images of flow cytometry analysis of Breg cell.

As shown in Figure 5, B cell and Breg cell ratios in the spleen of EAT rats in the M group were reduced in contrast with the N group. After selenium administration, B cell and Breg cell ratios in the spleen were enhanced in the Se group compared with the M group.

## 4. Discussion

The direct cause of AIT has not been elucidated until now; however, it is considered a combination of genetic susceptibility and environmental factors that leads to the loss of thyroid immunological tolerance. Environmental factors such as iodine play an important role in the induction and modulation of thyroid autoimmunity, inducing abnormal generation of autoantibody-producing B cells and autoreactive T cells and anomalous production of proinflammatory cytokines [27]. AIT is most frequently characterized as the dysfunction of the thyroid by the production of TGAb and TPOAb and the infiltration of monocytes and lymphocytes and results in gradual atrophy and fibrosis. EAT in animal models has been used to simulate human AIT by subcutaneous injection of PTG and fed with high iodine water according to previous studies [28, 29]. In the present study, compared with the N group, the EAT rats in the M group presented a number of lymphocytes and monocytes infiltration in the thyroid tissues and elevation of serum TSH, TPOAb, and TGAb concentration, indicating well simulation of AIT.

Selenium is an essential micronutrient with promising effects on the immune system [10, 11]. Thyroid is the organ with the highest selenium content in human being. Selenium supplementation is often suggested to AIT patients, in order to modify the inflammatory and immune responses of the thyroid. The effects of selenium supplementation on AIT were debated. Some meta-analyses have confirmed a suppressive effect of selenium supplementation on serum TPOAb and TGAb levels in AIT patients [12, 13]. However, other studies demonstrated the opposite results. Bonfig found that selenium supplementation with sodium selenite did not decrease TPOAb concentrations but could reduce TGAb concentrations in children and adolescents [30]. Karimi did not find any statistically significant difference in TSH and TGAb levels after sodium selenite treatment in patients with AIT [31]. Eskes conducted an RCT study in euthyroid TPOAb-positive women and concluded that six months selenium supplementation with sodium selenite had no effect on serum TPOAb and TSH levels [14]. The reasons why there were conflicting findings on autoimmunity of the thyroid by selenium supplementations in humans are complex, which might be possible owing to different intervention lasting time, different research sample sizes, or different age ranges, what is more, might be due to different selenium supplement type (such as inorganic sodium selenite or organic selenomethionine and selenium yeast) of each study. Our limited study observed that selenium yeast supplementation could relieve lymphocytes infiltration in the thyroid and relatively decrease serum TSH, TGAb, and TPOAb titers in the Se group compared with the M group, indicating its partially protective effects on EAT rats. Upon these results, the action of selenium yeast supplementation on AIT patients needs further study.

Furthermore, the mechanism of how selenium regulates B lymphocytes which produce TPOAb and TGAb in AIT was unclear. Therefore, our present study was aimed to detect the effects of selenium yeast on B cells and thyroid immunity in EAT rats. B cells play a key role in producing TGAb and TPOAb, which are crucial self-antigens responsible for apoptosis of thyroid follicular cells in AIT. Therefore, regulation of B cell activity might be an effective way to treat AIT. BAFF plays a critical role in regulating the maturation, proliferation, and differentiation of B cells and prolonging the survival of B cells [32]. Lin observed that serum BAFF levels were parallel to TPOAb and TGAb titers in Hashimoto (HT) patients [33]. In consistence with their results, our study found that BAFF levels were increased in EAT rats of the M group, consolidating that B lymphocyte activation, proliferation, differentiation, and autoantibody formation are involved in the pathological mechanism of autoimmune attacks towards thyroid tissue. In the Se group, BAFF levels were reduced in contrast with the M group, which indicated that selenium yeast might regulate the maturation, differentiation, and survival of B cells in the EAT model rats. On the other side, we observed that B cells ratio was reduced in the spleen of EAT model rats, although BAFF levels were increased, underlying that other immunocytes such as T cells might also involve in the complex pathology of AIT.

In the last years, an emerging role for Breg cells arouses more and more interests, which may inhibit the proinflammatory response, mostly by production of IL-10 cytokine, which exerts suppressive effects of autoimmunity [7, 8]. Actually, the characterization of Breg cells in AIT is still uncertain or controversial. Santaguida [34, 35] indicated the frequency of functional IL-10+ Breg cells was higher in HT patients than in healthy donors. On the contrary, Breg subset and IL-10 mRNA expression in splenocytes were decreased in studies conducted by Teng [9]. In consistence with the findings of Teng, we observed that Breg cell ratio in the spleen and IL-10 expression in thyroid tissue were reduced, reflecting suppressed activity of Breg cell, in our EAT model rats. In our limited experiment, when EAT rats were supplemented with selenium yeast in the Se group, IL-10 expression was increased in thyroid tissue, and Breg cell ratio in the spleen was elevated compared with the EAT model rats of the M group. These results suggested that selenium yeast might attenuate the autoimmune reaction of the thyroid in AIT through the regulation on Breg cell activity and IL-10 expression.

In summary, our experiment observed that serum BAFF was increased, while IL-10 expression in thyroid tissue and Breg cell ratio in the spleen were decreased, indicating the changes of B cell activation and Breg cell activity in EAT rats. Additionally, selenium yeast supplementation relieved lymphocytes infiltration and protected thyroid follicular cell morphology, lowered serum TGAB, TPOAb, and BAFF levels and IL-10 expression in thyroid tissue, and upregulated Breg cell ratio in the spleen of EAT rats, suggesting selenium yeast supplement could partially protect thyroid immune balance of EAT rats, which may be related to the modulation of B lymphocyte activity.

### 4.1. Strengths and Limitation of the Study

Our data indicated that organic selenium yeast supplement could be beneficial to attenuate immune imbalance in EAT model rats. However, there were some limitations in our study, one of which was that selenium concentration was not measured. Additionally, the present experiment only conducted in female rats, but the action of selenium yeast on male EAT model rats should also be observed. Moreover, these results should be demonstrated in other EAT models, such as in genetically susceptible strains of mice. Besides, the TPO and TG-specific B cell clones affected by selenium yeast supplementation need to be determined. Further studies should be conducted to interpret the effects and mechanisms of selenium supplementation on AIT in the future.

## Data Availability

The data used and/or analyzed during this study are available from the corresponding author upon request.
